# Artificial Vision Algorithms for Socially Assistive Robot Applications: A Review of the Literature

**DOI:** 10.3390/s21175728

**Published:** 2021-08-25

**Authors:** Victor Manuel Montaño-Serrano, Juan Manuel Jacinto-Villegas, Adriana Herlinda Vilchis-González, Otniel Portillo-Rodríguez

**Affiliations:** 1 Facultad de Ingeniería, Universidad Autónoma del Estado de México, Toluca 50130, Mexico; vmmontanos@uaemex.mx (V.M.M.-S.); avilchisg@uaemex.mx (A.H.V.-G.); oportillor@uaemex.mx (O.P.-R.); 2 Cátedras CONACYT, Ciudad de México 03940, Mexico

**Keywords:** trustworthy HRI, robot artificial cognition, HRIs in real-world settings

## Abstract

Today, computer vision algorithms are very important for different fields and applications, such as closed-circuit television security, health status monitoring, and recognizing a specific person or object and robotics. Regarding this topic, the present paper deals with a recent review of the literature on computer vision algorithms (recognition and tracking of faces, bodies, and objects) oriented towards socially assistive robot applications. The performance, frames per second (FPS) processing speed, and hardware implemented to run the algorithms are highlighted by comparing the available solutions. Moreover, this paper provides general information for researchers interested in knowing which vision algorithms are available, enabling them to select the one that is most suitable to include in their robotic system applications.

## 1. Introduction

Socially assistive robots (SARs) are a type of robot that interacts closely with people [1]. Due to their characteristics, they can communicate with and understand the activities and psychological state of a person, in order to respond in a positive way [2]. In addition, these robots can express feelings and emotions [1,3]; they are commonly used in tasks such as monitoring and caring for the elderly, supporting activities of daily living (ADL), controlling the behavior and health of patients, performing company work, and offering entertainment [4,5], besides helping with exercises and rehabilitation [6], among others. To provide assistance to people employing SARs, it is typically necessary to implement computer vision algorithms to identify the different objects in the environment, the user’s location, or the user’s face when involved in a specific activity. Computer vision is a growing area of study, in which constantly efficient algorithms, such as those for detection, tracking, and recognition, are developed to perform a task with minimal error and emulate human vision, which represents a challenge for different researchers.

Moreover, this paper deals with reviewing the literature on different computer vision algorithms used in SAR applications, highlighting the number of FPS corresponding to the velocity that each algorithm can process to determine if it can be used in real time, its performance presented in percentages, and the hardware/software used to obtain the results that the authors have reported.

### Methods

The method used to carry out this review of the literature is described next. Google Scholar, Elsevier, MDPI, and IEEE Explore databases were used to search for articles published in peer-reviewed journals, books, and conferences, within the interval period of 2010–2021. The keywords used for this review paper were: assistive robotics; face, body, and object tracking, and computer vision. The search was focused on selecting journals written in English, where the authors reported the experiments and results of their algorithms.

**Figure 1:**
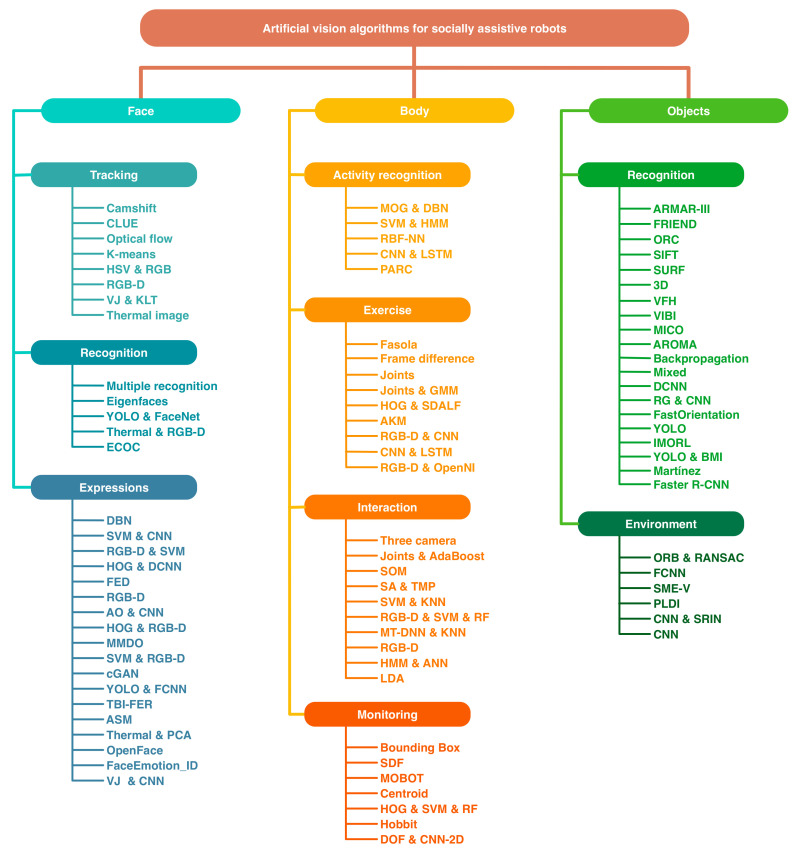
Classification of computer vision algorithms for SARs.

Previous related works were classified according to the SAR tasks shown in Figure 1. This classification was proposed due to the necessity for implementing computer vision algorithms focused on providing technological assistance for patients with mild cognitive impairment [7]. Furthermore, these algorithms were classified into three categories: face, body, and objects. Depending on the activities of interest, the algorithms can be grouped by subjects (for more details, see Figure 1).

The remainder of this paper is organized as follows: Section 2 presents the algorithms developed for face tracking. Section 3 includes the body tracking algorithms. Section 4 describes the algorithms related to object tracking. Finally, Section 5 provides the author’s discussions and conclusions.

## 2. Algorithms Used for Face Recognition and Tracking

Currently, thanks to the implementation of different algorithms in SAR applications, it is possible to perform different tasks, such as analyzing facial behavior (stress, anxiety, etc.) and facial attributes (gender, age, etc.), face editing (digital makeup), surveillance, sign language recognition, lip-reading, and human–computer/human–robot interaction [8,9], among others.

The literature shows that the most used algorithms for SARs are recognition, tracking, and facial expression. However, before working with the aforementioned algorithms, face detection must be carried out as a preliminary step. Locating the facial position within an image is essential because if an incorrect identification is made, the information provided will refer to a false face. Therefore, the features of the face that are used to carry out the consequent algorithms will also be incorrect.

The following sections show the algorithms that SARs use for facial tracking, recognition, and expression.

### 2.1. Tracking

Face tracking has become a challenge for computer vision, which involves locating one or more faces as an objective within a video for a defined period [8]. The computer vision algorithms that have been developed for face tracking are focused on tasks such as detection, locating landmarks, and recognition. Certain problems can affect the performance of these tasks: occlusion, deformation, and the position of the face when it is not in front of the camera.

Different algorithms have been developed to perform the task of face tracking, which extracts facial features (such as eyes, mouth, forehead, nose) from each frame to determine whether a face is present. For instance, due to its efficiency, one of the most widely used algorithms for face detection is the one proposed by Viola–Jones (VJ) [10,11], which combines the concepts of Haar-like features, integral images, the AdaBoost algorithm, and the cascade classifier. This algorithm presents a problem when it must correctly identify a face due to the occlusion perceived by the camera. To correct this problem, it is necessary to use an additional algorithm, such as the one reported by Putro et al. [8], who use the VJ algorithm to detect and extract facial features and then implement the Kanade–Lucas–Tomasi (KLT) algorithm to follow the face. This proposal is part of a robotic system used to accompany a person and can even locate a person and follow them through a room.

Another example is presented by Cilmi et al. [12], who developed a three-axis articulated neck for a robotic system that allows a natural interaction between the head of the robot and the person. The system can track the face in real time even when it is tilted or when the person is side-facing, as well as when the camera is in a lower or higher position. The authors implemented the VJ algorithm for face detection in conjunction with the KLT algorithm for face tracking in order to ensure its robustness. Moreover, Tanaka et al. in [13] presented the Coded Landmark for Ubiquitous Environment (CLUE) algorithm to control a robotic arm capable of helping a person to drink from a cup. The authors implemented a VJ algorithm to detect the face; then, for tracking, they used two cameras to compare 2D and 3D information to correct the position and distance of the face, and in this way, the robot can hold and bring the cup to the user’s mouth. On the other hand, Boccanfuso and O’Kane presented in [14,15] the Charlie robot to provide therapy to children with autism spectrum disorder (ASD). The authors were able to obtain information about the patient’s progress by employing a game that consisted of raising and lowering the hands. The robot also implemented the VJ and the Camshift algorithms for face detection and tracking, respectively. In addition, the patient’s gaze was used to determine whether he was paying attention to the robot; his hands’ positions were used to determine whether he was following the instructions given by the robot.

Another example of face tracking is presented by Perez et al. [16], who detected and tracked a patient’s face using an algorithm based on optical flow. Their eyes and mouth were detected even when there were changes in the intensity of light or the facial geometry. Using this algorithm, the authors could determine a person’s head movements, which were used as commands to control the movement of a mobile robot used as an autonomous wheelchair. Furthermore, Perez et al. presented another approach in [17] to detect the face by segmentation in the YCbCr color space of the frame. For face tracking, they calculated the centroids of the eyes and mouth by implementing the k-means algorithm to determine the position of the face and transform it into a command to move the same wheelchair. Moreover, Bhattacharjee et al. [18] proposed an algorithm for face detection and tracking based on segmentation in two color spaces, HSV (hue, saturation, value) and RGB (red, green, blue). First, the algorithm finds the regions that belong to the skin color to locate the face’s position within a frame; second, it converts this position into instructions and sends them to a robot, which uses them to move its wheels and track a person in a room in order to maintain his face in the center of the image.

On the other hand, Canala et al. [19] presented an algorithm to improve the interaction between the robot and the user; the authors implemented face tracking to describe gestures used by people without verbal communication (say yes or not). The NAO robot and Kinect vision system were used to implement deep maps to identify body parts, especially facial features such as the eyes, nose, and mouth.

Another approach was presented by Coşar et al. in [20], who used a thermal camera to identify the nose and forehead regions and to determine the respiratory and heart rate of older adults. This physiological monitoring system was integrated into an assistive robot for older adults at home. The authors first converted the thermal image into a binary image, then applied morphological operations to find the contours, and finally placed them over the facial landmarks to track them. These points were used to delimit a forehead and nose region; then, fast Fourier transformation on the thermal image was used to determine the respiratory and heart rate.

The above algorithms were found in the literature involving SARs that allow the tracking of the face. It has been found that most authors use the VJ algorithm to identify the face in an image. However, to correct problems and improve the performance, authors verify the presence of facial elements such as eyes, mouth, and skin color (see Table 1).

### 2.2. Recognition

Face recognition is another task that can be performed after a person has been detected in an image. This activity is commonly used for automatic safety systems. However, in the case of SARs, it can be used to determine whether a robot is interacting with the correct person. Face recognition is based on facial feature extraction; this information is stored in a database. Then, it is compared with new faces to identify whether a person is included in the database. In this section, the main algorithms used in state-of-the-art SARs are presented below.

An algorithm used for face recognition in SARs was proposed by Shoan in [21], which presents a robot capable of recognizing people to determine whether they have the authorization to be in a determined place. For this approach, the background subtraction algorithm detects first if there exists movement in the scene. Then, it determines whether a person is moving using the VJ algorithm and skin color detection. Finally, to corroborate whether the person is in the database, the Fisherface, eigenface, and the Local Binary Pattern Histogram algorithms are also implemented.

In addition, John et al. [22] proposed a model to improve the communication between a humanoid robot and user according to the type and degree of the user’s disability. Additionally, for facial recognition, they implemented the eigenface algorithm to recognize the user’s presence (from a dataset) and initiate a conversation. Another approach was presented by Ghita et al. [23], who proposed a new algorithm to recognize people and offer assistance with the Pepper robot in day-to-day environments such as public offices or stores. They used the You Only Look Once (YOLO) algorithm for face detection and implemented the FaceNet framework with a support vector machine (SVM) classifier for face recognition.

On the other hand, Coşar et al. in [24] presented an algorithm using a thermal and an RGB-D camera (depth image), performed by a robot to offer cognitive stimulation services for older adult users with mild cognitive impairments (MCI). The authors used the RGB-D camera information to detect the upper body; then, they used the thermal image to segment the head and face, and, combining both images, they could track, detect, and recognize people even when there were more people in a frame, achieving a recognition accuracy of over 70%, with four people detected at the same time.

Moreover, Chivarov et al. in [25] proposed an algorithm for the ROBCO 20 robot to recognize users with disabilities. The VJ algorithm was used for face detection. Then, the facial features were extracted utilizing the histogram-oriented gradient (HOG) algorithm. Finally, the multiclass error-correcting output codes (ECOC) algorithm and the user’s voice were utilized to recognize the user.

According to the algorithms mentioned above, employed for the recognition of faces, it has been found that some authors employ algorithms based on geometry. However, algorithms that use classifiers, such as eigenface, Fisherface, and Local Binary Pattern Histogram (LBPH), are employed more frequently because authors can extract the characteristics of the face to generate a model with the faces that they wish to recognize (see Table 1).

### 2.3. Facial Expressions

In computer vision, another task related to the face is the detection of facial expressions. In this case, SARs use the information to determine the different moods of people, in order to respond adequately to different situations. Usually, the moods that are detected are sadness, happiness, stress, and depression. This section presents the algorithms that have been used in SARs to detect facial expressions.

For example, Ramirez et al. [26] presented the ONO robot, which detects early autism in children. This robot detects the face using HOG and a maximum margin object detection model (MMDO) trained to detect faces, which classifies as known or unknown using a convolutional neural network (CNN). Furthermore, it uses the Conditional Local Neural Fields (CLNF) algorithm to place marks on the face, to analyze the emotions that a child presents during therapy. Another approach is the one presented by Ruiz et al. in [27]: the authors proposed to recognize emotions by analyzing facial expressions using a combined algorithm that implements CNN for feature extraction and SVM for classification. In addition, a database with faces and different moods is used; it is worth mentioning that this approach does not use any algorithm for face detection. The NAO robot uses the trained algorithm to recognize the following moods: anger, disgust, fear, happiness, indifference, sadness, and surprise.

Moreover, Ruiz et al. [28] presented an algorithm to recognize and react to six emotions (anger, disgust, fear, happiness, sadness, surprise) to be used in the NAO robot as an accompaniment for children with ASD. They used the HOG model for face detection; then, facial features were extracted and classified with a deep CNN. Furthermore, Babu et al. [29] presented a multi-modal algorithm for NAO robots that consists of obtaining face and body expressions to determine the user’s emotional state when interacting with a robot: (a) the Adam Optimizer (AO) algorithm detects and extracts the facial features (eyes, eyebrows, and mouth); (b) the body’s position is extracted utilizing an RGB-D camera; (c) combining the face and body information into a CNN, expressions can be classified as positive, neutral, or negative emotional states, allowing the robot to behave appropriately.

Moreover, Deng et al. in [30] performed an algorithm conditional generative adversarial network (cGAN) based on three discriminators (Di, Da, and Dexp) to determine emotions through the facial expressions of a person that interacts with a robot. The authors used a multi-task cascade convolutional neural network (MTCNN) and OpenFace to detect the face. In addition, they implemented a generator G to extract the facial features and generate a synthetic image with more discriminative features for expression recognition.

Additionally, Benamara et al. [31] performed an algorithm for a sociable robot to recognize emotions when interacting with a person in real time. The algorithm detects the face by employing a YOLO framework; then, it converts the resulting image in grayscale, normalized in the range [0, 1]. In the next step, a CNN is used for feature extraction through edge and shape detection. Finally, the authors implemented a fully convolutional neural network (FCNN) to perform four models to classify emotions.

In addition, Ilyas et al. [32] proposed the traumatic brain injured facial expression recognition (TBI-FER) algorithm to enhance social interaction and assist trainers and physiotherapists of traumatic brain injured people using the Pepper robot. The algorithm implements the Supervised Descent Method (SDM) to detect the facial landmarks and track them. Then, a pre-trained CNN with a VGG-16 model is implemented to extract the facial features, and a Long Short-Term Memory (LSTM) neural network is used to exploit the spatio-temporal information, allowing the Pepper robot to detect six different emotional states (happiness, sadness, fear, anger, surprise, and disgust) plus a normal state.

Another example is presented by Ramis et al. in [33]. The authors proposed an algorithm to recognize the emotions of people using the NAO robot, employing a game: (a) the VJ algorithm carries out face detection; (b) facial landmarks are located to calculate the center of each eye and the distance between them; (c) a CNN processes the image to determine whether the expression relates to happiness, sadness, disgust, anger, surprise, fear, or indifference.

On the other hand, Sridhar et al. presented in [34] the E-bot vision system, which was developed using a Raspberry Pi. The system was used to identify the mood of a person by employing Google Cloud Vision API and facial expression detection (FED); both algorithms used a CNN and internally detected the face through facial feature extraction and the classification of the different moods of a person. Additionally, with the proposed approach, the authors obtained an accuracy of 60 % to identify whether the person was angry, scared, happy, sad, surprised, or in disagreement.

Pour et al. [35] presented an algorithm for a robot to support the detection of emotions and to be able to respond appropriately to those that a child with autism expresses during therapy. To carry out this work, the authors used a Kinect sensor to extract facial features in a set of data points (point cloud); then, 18 points were identified on the face following the facial action coding system. Moreover, to classify and determine the distance between the points assigned to the face, the fuzzy c-means method was implemented. Another robot that uses a point cloud to offer therapy is the Mini robot presented by Castillo et al. in [36], which helps people with mouth articulation problems when speaking. It uses a computer vision system based on Kinect, which extracts the posture of the mouth and, using the support vector machine (SVM) algorithm, verifies that the posture of the mouth corresponds to the exercise being performed; in this way, it detects errors to provide visual and audible help to the person. Moreover, Silva et al. [37] presented an algorithm for emotion recognition to train the RoboKind Zeno R50 robot to assist children with ASD. They used the Intel RealSense sensor to acquire a deep map and extract facial features. Then, they used the SVM algorithm to identify the user’s emotions as anger, fear, happiness, sadness, surprise, or indifference.

Another example of computer vision in SARs is the work of Pino et al. [38], which uses an algorithm based on points, which they place on the eyes, nose, mouth, eyebrows, and contours of the face. Then, they join the points to form geometries, to which they extract their area, and in this way, they can analyze the state of mind and recognize whether a person with a mild cognitive impairment has anxiety or depression.

Bayesian networks have also been used for the recognition of facial expressions; for instance, Cid et al. in [39] presented a proposal for a Dynamic Bayesian Network (DBN) to identify the facial expressions of a person and determine their mood, to train a robot to mimic the facial expressions. The VJ algorithm detects the face; then, the eyes and mouth are treated with a Gabor filter to determine the edges of the face extracted, which are shared with the DBN.

Another proposal was offered by Meghdari et al. [40] using the R-50 Alice robot for emotion recognition through the Kinect sensor. The authors implement the deep map for facial feature extraction, comparing a database to determine whether the emotional state is happiness, sadness, anger, surprise, disgust, or fear. The Alice robot responds with another emotional state selected by a fuzzy algorithm to improve the interaction between robot and user.

Meanwhile, Fang et al. [41] proposed an algorithm to offer help to disabled people when eating, using a robotic arm, an RGB camera, and a depth sensor. First, they detect the face with the depth sensor; then, the HOG algorithm is applied to identify the facial landmarks and determine the position and depth of the mouth. Finally, the information is converted into motion commands so that the robotic arm can take the food to the user’s mouth.

Furthermore, Goular et al. in [42,43] proposed an algorithm for the N-MARIA robot to recognize children’s emotions in order to offer a better child–robot interaction utilizing thermal images. The authors used the VJ algorithm to detect the face; then, the thermal image was used to identify the eyes, nose, forehead, cheek, perinasal, and periorbital region. In the next step, the principal component analysis (PCA) algorithm extracted the facial features. Finally, the Latent Dirichlet Allocation (LDA) algorithm was implemented to classify the emotion detected as either disgust, fear, happiness, sadness, or surprise.

On the other hand, Jain et al. [44,45] developed a model to determine the engagement level in SAR interventions for children with ASD while they are playing mathematical games. The authors used OpenFace because this framework provides high face detection confidence value, eye gaze direction, head position, and facial expression features. In addition, the authors used audio features; this information was classified with the PCA algorithm to determine whether a child was engaged or disengaged with the robot interventions.

Lamas et al. in [46] proposed the FaceEmotion_ID algorithm for the emotion recognition system of the NAO robot during the monitoring of patients with MCI. In addition, the authors implemented speech recognition and combined it with the facial expression to determine whether the patient had completed their activity and propose another one. In this way, the patient is kept busy, completing daily life activities.

In the above paragraphs, the algorithms for determining people’s facial expressions have been presented. Most of the proposals have been developed to determine the state of a person’s mood; this allows SARs to perform accompaniment work or offer help during the therapy of children with autism. In addition, to determine the mood using computer vision, most authors have selected classifiers based on neural networks, either with a normal CNN or through a framework such as OpenFace. Similarly, neural networks are applied in 2D and 3D images (see Table 1).

In this section, the algorithms that have been used with SARs for tasks involving the face were presented. Table 1 shows a comparison between these algorithms, which can be used by other researchers to choose the most suitable algorithm for their applications; it also permits researchers to identify whether an algorithm needs to be improved or a new one needs to be developed.

**Table 1:** Comparison of face tracking algorithms.

Algorithm	PERF	Speed	Hardware and Software	Application
Camshift (2010) [14]	85%	real time	OpenCV	Tracking
CLUE (2010) [13]	-	-	MANUS Assistive Robotic Manipulator (ARM) and OpenCV	Tracking
Optical Flow (2013) [16]	-	real time	-	Tracking
DBN (2013) [39]	-	25 FPS	2.8 GHz Intel(R) Core(TM) i7 CPU and 4GB RAM running using GNU/Linux Ubuntu 10.10	Expressions
K-means (2013) [17]	-	10 FPS	Computer at 2.4 GHz	Tracking
HSV & RGB (2015) [18]	99%	real time	2 GHz Intel Core2Duo and 2 GB RAM. Programming in Matlab	Tracking
Multiple face recognition (2015) [21]	91%	real time	2.3 GHz i5 and 4 GB of RAM running using Windows 7 (×64)	Recognition
SVM & CNN (2016) [27]	96.26%	real time	NAO robot	Expressions
RGB-D (2016) [19]	73.33%	real time	NAO and Wifibot robots, a Kinect v2 sensor, and two conventional laptops	Tracking
Eigenfaces (2016) [22]	-	-	InMoov Robot, an open-source robot that can be printed, and conventional PC	Recognition
RGB-D & SVM (2017) [37]	93.6%	real time	RoboKind Zeno R50 (ZECA) robot	Expressions
HOG & DCNN (2018) [28]	99.14%	real time	NAO robot	Expressions
VJ & KLT (2018) [12]	-	10 FPS	PC whit GPU does not specify which	Tracking
FED (2018) [34]	80%	offline	Raspberry Pi 3 Model B+	Expressions
RGB-D (2018) [40]	90%	real time	The R-50 Alice (Mina) robot	Expressions
RGB-D (2018) [35]	93.2%	real time	The R-50 Alice (Mina) robot	Expressions
AO & CNN (2018) [29]	91%	real time	NAO robot and Kinect sensor	Expressions
VJ & KLT (2018) [8]	90%	28.32 FPS	Intel Core i5-6600 CPU @ 3.30 GHz, 8 GB RAM	Tracking
HOG & RGB-D (2018) [41]	-	4 FPS	Intel Core i5	Expressions
MMDO (2018) [26]	-	27 FPS	Two workstations with GTX960 GPU, and one workstation with GTX580 GPU, all with a processor of Intel Core i5	Expressions
SVM & RGB-D (2018) [36]	88%	offline	Mini robot	Expressions
Thermal image (2018) [20]	-	real time	Enrichme robot and Optris PI-450 camera	Tracking
YOLO & FaceNet (2018) [23]	-	30 FPS	Pepper robot	Recognition
cGAN (2019) [30]	74.80%	offline	Workstation with GeForce GTX 1080Ti GPU	Expressions
Thermal & RGB-D (2019) [24]	90.59%	real time	THIAGo robot, the Ambient Intelligence System (AIS), the Networked Care Platform (NCP), and Optris PI450 thermal camera	Recognition
YOLO & FCNN (2019) [31]	72.47%	real time	PC with i7 CPU processor and Nvidia Tesla K80 GPU	Expressions
TBI-FER (2019) [32]	88%	real time	Pepper robot	Expressions
ASM (2019) [38]	97.67%	real time	NAO robot and PC with AMD Geode with 500 MHz CPU, 256 MB SDRAM and 1 GB flash memory	Expressions
Thermal & PCA (2019) [42]	85.75%	2 FPS	N-MARIA robot	Expressions
OpenFace (2020) [44]	90%	real time	Kiwi robot, conventional camera and tablet	Expressions
FaceEmotion_ID (2020) [46]	-	-	NAO robot	Expressions
ECOC (2020) [25]	90%	-	ROBCO 20 robot whit intel Core i7-8705G CPU	Recognition
VJ & CNN (2020) [33]	82%	real time	NAO robot	Expressions

Performance (PERF) is the percentage of frames that have followed a face correctly. Speed is the number of FPS that can be processed. Hardware is the computer setup that was used to perform the tests. Software is the development platform used. The application refers to the task for which the algorithm is intended. Where data were not available, this is represented by a (-).

On the other hand, in this section, it can be appreciated that the most popular algorithm used for face detection is the one proposed by Viola–Jones. Due to its efficiency, a few authors have included a correction stage to eliminate the false faces detected. Several algorithms have been developed regarding the facial tracking task, which often involves finding characteristic features of the face, such as the eyes, mouth, and skin color, without any predominant features. Likewise, artificial networks are mostly implemented to develop algorithms for the recognition of expressions. In contrast, for face recognition, the eigenface, Fisherface, and Local Binary Pattern Histogram algorithms are the most used. The author in [21] implemented a multiple face recognition algorithm and reported performance of 99.87%.

In the most recent research of the last five years, the authors implemented frameworks such as OpenFace [44], YOLO [31], and FaceNet [23], because these are frameworks that include robust algorithms that can achieve performance of up to 90%. However, they require computer equipment with sufficient computing power to obtain the best performance.

## 3. Algorithms Used for the Body

For SARs to offer aid to people, different algorithms have been developed that allow them to recognize body parts and their movements. In this way, SARs can recognize the activities of daily living in order to support users or offer personal training to carry out their exercise routine, or they can be used for monitoring to determine the user’s health status or understand non-verbal communication. This section presents the algorithms that have been used with SARs to recognize parts of the human body.

### 3.1. Activity Recognition

One of the most critical tasks performed by SARs is activity recognition; with this, it is possible to determine whether an elderly or disabled person can carry out an activity alone or whether they require assistance. Algorithms used for activity recognition implemented by SARs are presented below.

Firstly, McColl and Nejat proposed [47] an algorithm to determine older adults’ affective body language when interacting with a robot. The authors implemented a Kinect with the Mixture of Gaussians (MOG) algorithm to detect the body and convert it into a 3D geometrical model. Then, the 3D skeleton model was extracted to be tracked by the DBN algorithm. Finally, the Adaboost algorithm was implemented to classify the body language using forward/backward head positions, opening and closing the arms, and bowing and stretching the trunk.

On the other hand, Wu et al. in [48] performed an algorithm for an assistive robot to recognize human activities. They implemented the kinect sensor to extract the body structure and joints. Then, they combined the SVM algorithm and Hidden Markov Model (HMM) to train a model to classify the activities.

Another example is presented by Rossi et al. [49]; the authors proposed an algorithm for the Pepper robot to recognize the ADLs. They used an RGB-D camera to extract the joints in the body. A double-layer network (CNN and LSTM) was implemented to recognize the following activities: brushing teeth, chopping, drinking water, opening pill container, relaxing on the couch, rinsing the mouth with water, stirring, talking on the couch, talking on the phone, wearing contact lenses, working on the computer, and writing on a whiteboard. Additionally, Massardi et al. in [50] presented the PARC algorithm for an assistance robot to recognize and help people with cognitive or physical disabilities to perform ADLs: (a) an RGB-D camera was implemented to extract the body and objects utilizing the YOLO framework; (b) they calculated whether an object was in the person’s hand to identify the activity; (c) they used a particle filter algorithm to plan the sequence of tasks that the user must perform to complete the activity.

Nevertheless, Ravichandar et al. presented in [51] an algorithm for the Baxter robot to infer the intended actions of the user in order to collaborate with them during the performance of a task. The authors extracted the skeleton by employing a Kinect sensor and used the Kalman filter to obtain the joints’ positions, velocity, and acceleration. Then, a radial basis function neural network (RBF-NN) was trained. Finally, when a new measurement was available, the E-M algorithm was implemented to determine which movement the person would perform.

The algorithms described above are those that SARs use to determine the activity that a person performs. It has been found in the literature that there is no single algorithm used most frequently. However, it can be highlighted that the described algorithms are based on determining a person’s activity using the movement of body parts to compare them with known patterns, as well as identifying objects that a person manipulates in order to perform different activities (see Table 2).

### 3.2. Exercise

Another available SAR function is to offer assistance as a personal trainer for users during their rehabilitation exercises, in order to improve their health. One example of such an algorithm is presented by Fasola et al. in [52,53], who implement a robotics platform as a personal exercise trainer for older adults through games: (a) some threshold is defined to binarize the image; (b) the hand locations are determined by examining the extreme points of the body pixels in the segmented image; (c) the elbow position is estimated to provide the arm angles. Moreover, Gadde et al. in [54] presented a robot capable of indicating and motivating a person to perform exercise. Hand detection and tracking by the frame difference algorithm are implemented. Then, the robot instructs the user to raise or lower their arms. Moreover, Gorer et al. in [55] presented an algorithm for the NAO robot to help people with MCI to perform exercise. In addition, they implemented a Kinect sensor to extract the body skeleton; then, the joints’ angles were used to determine whether the user performed the exercise correctly. Otherwise, the NAO robot motivated the person through sounds and examples of how the exercise should be performed. In the same way, Fang et al. [56] proposed an algorithm for a mobile robot to assist in rehabilitation routines as a monitor. They implemented a Kinect sensor to extract the skeleton. Then, the arm positions were computed to track the hands. Finally, they classified the movements according to six gestures (upward sliding, downward sliding, left, right, clockwise rotation, and counterclockwise rotation).

Furthermore, Martinez et al. [57] proposed an algorithm for the Pepper robot to promote and monitor older adults’ physical activity in their living environments. They used an RGB-D camera to extract the skeleton joints, and then a double-layer network (CNN and LSTM) through the OpenPose framework recognized up to 24 different exercises. Tanguy et al. in [58] proposed an algorithm for the Poppy humanoid robot to assist in rehabilitating upper body exercises. The authors implemented Gaussian Mixture Models (GMM) to identify the movement tolerated variance for each joint and timeframe. In addition, the robot provided feedback, presenting and explaining the exercise to the patient. Another example is presented by Gross et al. in [59,60], who proposed an algorithm for a companion robot to motivate patients to start, continue, and repeat their exercises: (a) the HOG algorithm is implemented to detect the upper body; (b) the Symmetry-Driven Accumulation of Local Features (SDALF) algorithm is used to extract body features; (c) the motion is tracked to determine whether the patient has correctly performed the exercise.

On the other hand, Lotfi et al. in [61] proposed an algorithm for SARs to provide visual and audio feedback with facial expressions and motivational words depending on the user’s performance when carrying out exercise. First, the skeleton and joints are extracted utilizing a Kinect sensor; then, the joint coordinates and their angles are computed. Finally, the Angular Kinematics Model (AKM) is used for joint tracking. Ma et al. in [62] developed an algorithm for SARs to recognize the upper limb posture in the rehabilitation and assessment of stroke patients. The authors detected the arms by combining a depth image with a color image. Then, a CNN was implemented to extract the position of four joints (shoulder, elbow, wrist, and hand) in order to track the arms.

Among the algorithms developed to verify whether a person is exercising, it was found that most of the authors chose to extract the skeleton of the user by employing the Kinect sensor. Later, they determined whether there was a movement by measuring the angles between the joints. These algorithms have been used for patients who can perform exercises that involve the upper body (see Table 2).

### 3.3. Interaction

The correct interaction between robots and people is necessary for SARs to behave appropriately and recognize body expressions, because people commonly use non-verbal communication when speaking or expressing moods. This section presents the algorithms used to recognize body expression implemented by SARs. For example, McColl et al. in [63] presented an algorithm to determine the body expression when a robot interacts with a person. The algorithm uses information (time of flight, thermal, and 2D) from three cameras to detect and track the trunk, arms, and head; then, the position is classified by the body trunk lean direction (upright and forward) and the orientation patterns, like towards, neutral, or away from another person. Another example is presented by Guler et al. in [64,65]; authors used an RGB-D camera and laser sensor to recognize the human gestures when a robot interacts with a person. The algorithm extracts the skeleton and estimates the joint angles using geometry. Finally, the HOG, Hall-of-Fame (HOF), and Markov-Block-Hankel (MBH) algorithms are combined to classify eight gestures: help, want to stand up, perform a task, want to sit down, come closer, come here, let’s go, and park.

On the other hand, Ge et al. in [66] proposed an algorithm for a social robot to assist a child with ASD in therapy. First, an RGB-D camera is implemented to extract the skeleton in order to calculate the leaning angle, planar distance to therapist, mean joint to joint distance, the distance of joints traveled within task ball, mean joint coordinates, mean joint distance to task, and mean joint to joint distance, to classify them into engagement or disengagement during a game. Moreover, Guneysu et al. in [67] presented an algorithm for the FACE robot to detect social and emotional cues. The authors implemented the Scene Analyzer (SA), a framework based on an RGB-D camera to extract the body skeleton, facial expression, age, and gender of the user. In addition, they incorporated the Touch-Me Pad (TMP) framework to monitor the physiological parameters correlated to human affective states. Instead, Marinoiu et al. in [68] proposed an algorithm for a remote-controlled robot to engage a child with autism in emotion learning therapy. The authors implemented a Kinect sensor to extract the joints of the body and then a Multi-Task Deep Neural Network (MT-DNN) for bodies with occlusion was used to estimate the positions of the joints in 2D and 3D. Finally, a KNN classifier and a CNN were trained to determine the action performed by the child.

In addition, Gurpinar et al. in [69] presented an algorithm for the Robovie R3 Robot to be able to recognize autistic children’s hand and head gestures during therapy. A Kinect sensor is also implemented to extract the upper body skeleton. Then, the HMM and ANN algorithms are combined to determine the body posture. Furthermore, Rodríguez et al. [70] proposed an algorithm for the Pepper robot to recognize the actions (say hello, shake hands) that a person performed to improve human–robot interaction. Their proposal consists of implementing the Kinect sensor to extract the skeleton using the OpenPose and the HOG algorithms. Then, the LDA classifier is used to identify pose patterns.

Moreover, Kurien et al. in [71] proposed an algorithm for the control of a robot to carry out a construction task (to stack bricks); this aims to prevent people from being exposed to the risks that can arise in a construction site. The authors implemented a Kinect sensor to calculate the joint positions of the worker’s arms and hands to track their movements. Then, this information was interpreted as input commands to train the robot arm, which was the one that performed the construction task.

Another proposal is performed by Tuyen et al. in [72], who presented an algorithm to increase the user’s attention in order to enhance their engagement with and empathy for SARs. First, they used a Kinect sensor to extract the skeleton. Then, the joints’ positions were calculated by employing a covariance descriptor. Finally, a self-organizing map (SOM) with a k-means classifier was trained to group 12 different body expressions. Furthermore, Adama et al. in [73] proposed an algorithm for a social robot to learn human activities and improve human–robot interaction in living environments. The authors extracted the skeleton from RGB-D images; then, the joints were extracted to identify 13 different ADLs utilizing SVM and KNN algorithms. Alternatively, the same authors in [74] proposed a modification to their mentioned algorithm to include Random Forest (RF) to classify human activities. On this occasion, they used four different activities (brushing teeth, picking up an object, sitting on a sofa, and standing up) to test their algorithm.

Body part detection and expression recognition have been used during therapy for children with autism; in this way, the behavior of a patient can be determined. In the case of SARs, this information is used to react appropriately and improve their interactions with people. Regarding the algorithms, it was found that most of the authors used the skeleton of the user to determine the gestures performed with their hands and head. Moreover, it was found that neural networks are applied mainly in 2D and 3D images to classify gestures (see Table 2).

**Table 2:** Comparison of body tracking algorithms.

Algorithm	PERF	Speed	Hardware and Software	Application
Fasola (2010) [52]	-	20 FPS	The torso comprises 19 controllable degrees of freedom and a Pioneer 2DX mobile base. OpenCV	Exercise
Three camera (2011) [63]	-	real time	Dell workstation with Intel Xeon 3.2 GHz CPU and 2.0 GB RAM utilizing Matlab	Interaction
Frame Difference (2011) [54]	-	real time	Humanoid RoboPhilo Robot. 32-bit PC with Windows XP, intel Core 2 Duo CPU, 4 GB of RAM	Exercise
MOG & DBN (2014) [47]	93.6%	-	Brian 2.1 robot	Activity
SVM & HMM (2014) [48]	98.11%	real time	-	Activity
Bounding Box (2015) [75]	92.6%	off-line	Conventional workstation with Ubuntu	Monitoring
Joints (2016) [55]	-	real time	NAO robot, Kinect sensor, and conventional laptop	Exercise
Joints & GMM (2016) [58]	-	real time	Poppy robot, Kinect sensor, and open source	Exercise
Joints & AdaBoost (2016) [66]	97%	-	Kinect sensor	Interaction
RBF-NN (2017) [51]	-	20 FPS	Standard desktop computer with Intel i3 processor and 8 GB of RAM. Coded in Matlab 2014a	Activity
SOM (2017) [72]	91.1%	offline	Not described	Interaction
HOG & SDALF (2017) [59]	95%	real time	Robot platform ROREAS	Exercise
SA & TMP (2017) [67]	-	real time	FACE robot and Kinect sensor	Interaction
SDF (2017) [76]	97.4%	24 FPS	Workstation with GPU	Monitoring
MOBOT (2017) [77]	93%	real time	MOBOT robot	Monitoring
Centroid (2017) [78]	-	offline	Mobile robot, Kinect sensor, Arduino mega board and standard laptop	Monitoring
HOG, SVM & HSV (2017) [79]	-	real time	Not described	Monitoring
SVM & KNN (2017) [73]	99.73%	offline	Not described	Interaction
Hobbit (2018) [80]	-	real time	Hobbit Robot	Monitoring
RGB-D, SVM & RF (2018) [74]	92.32%	offline	Not described	Interaction
AKM (2018) [61]	-	real time	Windows 64-Bit PC with 4 GB RAM, Kinect Sensor (Version 2), its Windows Adapter, telescopic robotic for Double Robotics and Kinect v2	Exercise
RGB-D & CNN (2018) [62]	97.8%	real time	PC with i5 processor, GeForce GTX1060 GPU, and Kinect v2	Exercise
MT-DNN & KNN (2018) [68]	50%	off-line	Not described	Interaction
RGB-D (2018) [71]	-	real time	Kinect sensor and Workstation not described. Simulated robot hardware	Interaction
CNN & LSTM (2018) [49]	-	-	Pepper robot	Activity
CNN & LSTM (2019) [57]	99.87%	-	Pepper robot	Exercise
RGBD & OpenNI (2019) [56]	95%	30 FPS	Mobile robot, conventional workstation, and Arduino board	Exercise
HMM & ANN (2019) [69]	97.97%	real time	Robovie R3 Robot	Interaction
DOF & CNN 2D (2020) [81]	-	5 FPS	iRobot Roomba mobile base, an Apple iPhone, and an Intel i5 processor mini PC	Monitoring
PARC (2020) [50]	80%	real time	Off-board ASUS Zenbook with an Intel Core i5-6200U CPU, 8 GB RAM, an Intel RealSense D-435 RGB-D camera, and a Turtlebot 2e mobile platform1. The software is developed in C++ with OpenCV libraries and YOLO V3	Activity
LDA (2020) [70]	90%	10 FPS	Pepper robot	Interaction

Performance (PERF) is the percentage of frames that have followed a face correctly. Speed is the number of FPS that can be processed. Hardware is the computer setup used to perform the tests. Software is the development platform used. The application refers to the task for which the algorithm is intended. Where data were not available, this is represented by a (-).

### 3.4. Monitoring

Another function of SARs is to act as a personal trainer for patients who have had an accident or need to increase their physical activity. The SARs can assist in ensuring that the exercises are correctly performed and recording the user’s progress during the exercise sessions.

An algorithm used in SARs for monitoring people is the one proposed by Dimitrov [75], which presents an algorithm for the PARbot robot for fall detection. They implement an RGB-D camera to extract the skeleton; then, the authors estimated the position of the head, torso, neck, shoulders, elbows, hands, hips, knees, and feet. Finally, the entire skeleton was reduced to a bounding box defined by its width, depth, height, and the vertical position of the torso to determine if the person had fallen.

On the other hand, Vasileiadis et al. in [76] proposed an algorithm for an assistive robot to track the body pose, which is part of the Horizon 2020 program to develop the RAMCIP robot. First, the skeleton and joints are extracted utilizing a depth camera. Then, it matches with a joint body template to determine the body orientation. Finally, a signed distance functions (SDF) model is computed to correct and track the body position. Moreover, Koumpouros et al. in [77] presented the MOBOT robot, which is capable of monitoring human performance and postural stability to detect falls and to recognize the human physiological state. The authors used an RGB-D camera for body pose estimation and a LIDAR sensor to detect and avoid obstacles. Nevertheless, Ahmad et al. in [78] presented an algorithm for a mobile robot to follow a person around a room with obstacles. The authors used a Kinect sensor to detect the human body, utilizing a skeleton to determine the center of mass. Finally, they used the body location to orient a mobile robot to track a person.

Another example is presented by Htwe et al. in [79], who developed an algorithm for a social robot to monitor students at school: (a) first, the HOG and SVM algorithms are implemented to detect the body; (b) an HSV color filter is applied to segment the upper and low body; (c) a Kalman filter is used to determine the body position even with occlusions. The robot uses the position to track students.

However, Bajones et al. in [80] proposed an algorithm for the Hobbit robot to reduce the risk of fall and injury in elderly people. The authors implemented a depth sensor to detect the joints of the skeleton and recognize the body gestures. For fall detection, one thermal camera is used together with the inclination of the robot’s head to see if there is a person on the floor.

In addition, Chin et al. in [81] developed an algorithm for a mobile robot to detect falls in older adults living alone at home. First, spatial and temporal information is computed using a dense optical flow (DOF) algorithm. Then, a modified CNN for 2D images is implemented to track and determine the person’s position relative to the ground.

Monitoring is one of the most crucial tasks that SARs can perform because they are used for people who live alone and are at risk of falls. Most authors have chosen to use the complete skeleton of a person using 3D imaging. Finally, the position of the person is calculated with an algorithm that can be based on the geometry of the body or on previous models (see Table 2).

In this section, the algorithms used to recognize body parts have been presented. Table 2 shows a comparison between these algorithms; authors interested in developing an SAR can use the information to determine whether the algorithm can be implemented, improved, or a new one developed.

In addition, in this section, it can be appreciated that there is no popular algorithm used for activity recognition. However, authors implemented body part recognition to determine the sequence of a person’s postures. With these algorithms, they obtained performance of up to 98.11% [48] using a Kinect sensor and SVM and HMM to classify activities. Nonetheless, other authors in more recent works did not report the performance that they achieved.

On the other hand, among the algorithms that were implemented to monitor patients during exercise, the authors of [57] reported 99.87% performance, using a CNN and an LSTM deployed in a Pepper robot with a Kinect sensor. However, other authors reported performance greater than 95%; they used other robotic platforms such as the NAO robot and the Poppy robot. Furthermore, most of the authors described their algorithms as being suitable to perform in real time.

Regarding the interaction, the algorithm presented in [69] has the best performance (97.97%); the authors implemented the HMM and ANN algorithms to determine body postures during therapy for children with autism. Moreover, the authors reported that the algorithm runs in real time. Moreover, their tests were performed on the Robot Robovie R3.

For algorithms for monitoring, the most popular application is fall detection. However, the algorithm proposed in [76] to recognize the body postures of ADLs has the best performance reported (97.4%), employing an SDF model. The authors used a workstation with a GPU to achieve this performance. However, they did not specify the hardware and software equipment used.

## 4. Algorithms Used for Objects

Another challenge for SARs is the detection and recognition of objects. This ability allows them to support disabled people, older adults, and healthy people to perform their ADLs. Different algorithms have been developed for object recognition to integrate the interaction modules of SARs, which allow the performance of tasks such as carrying things, picking things up, avoiding obstacles, and navigating.

### 4.1. Algorithms Used for Object Recognition

The systems, also known as Assistive Robotic Manipulators (ARMs), are primarily used to recognize and locate some objects in an environment. ARMs consist of a mobile or static robotic arm and a computer vision system to help upper-body-disabled people to complete ADLs. The main tasks performed by these systems are: picking up food and drinks from a table and bringing it to the person’s mouth, picking up objects from the floor, and recognizing objects to give to a person. This section presents the algorithms used to recognize and locate objects implemented by SARs.

One example of such an algorithm is presented by Yamazaki et al. in [82], who proposed an algorithm for an ARMAR-III robot capable of recognizing and manipulating kitchen objects to make a salad: (a) an edge and simple 3D models are implemented to determine the location of the cutting board; (b) background subtraction and color segmentation are used to find the vegetables, knife, and bowl; (c) the contours of the vegetables are computed to establish their positions. Moreover, Martinez and del Pobil in [83] proposed a hybrid algorithm for the T040 robot capable of detecting and recognizing objects from visual input in ordinary environments. The authors implemented three algorithms for object detection: color space (Lab) for segmentation, Gabor filters for shape recognition, and frame difference and background subtraction for motion detection. Then, a statistical combination of similarity likelihood was performed to classify objects.

In addition, Maymo et al. in [84] proposed the FastOrient algorithm for the UR10 Universal Robot to be able to determine the orientation of the target object to be picked up. First, the image is acquired and transformed to grayscale. Later, it is converted to the HSV color space and then segmented by means of the threshold algorithm. Finally, the axes are computed to determine the position of the object. Moreover, Martinez et al. in [85] proposed an algorithm for assistive robots capable of picking up objects from the ground to help people at home. The authors implement a Kinect sensor to extract a point cloud to analyze the 3D scene. Then, they convert it to the Lab color space to match the depth to the segment. Next, the edges are computed to separate objects from the floor. Finally, the contact points are estimated to provide the robot with the location of the object.

On the other hand, Natarajan et al. in [86,87] proposed an algorithm for the FRIEND (Functional Robot with Dexterous Arm and User-Friendly Interface for Disabled People) robot for recognizing and reconstructing real-world objects corresponding to ADL scenarios. The authors implemented stereo vision to perform color segmentation and deep discontinuity between regions to identify all the objects’ regions. Then, basic shapes (cuboid and cylinder) are used to reconstruct and locate the objects. Furthermore, Yamazaki et al. in [88] implemented stereo vision to propose the Optical Character Reader (ORC) algorithm for an assistive robot to be able to separate garbage into two categories (combustible material and plastic bottle). First, edge and contour detection algorithms are implemented to identify the characters and symbols that objects have. Then, a Hough transform is used to calculate the orientation of the objects. Finally, the location of the objects is computed so that the robot can take them.

In contrast, Zhang et al. in [89] proposed an algorithm for an assistive co-robot to offer aid to recognize objects in factories. The authors implemented the scale-invariant feature transform (SIFT) algorithm to extract the features of the object; then, a hierarchical k-means algorithm was used for the classification stage. In addition, Leroux et al. in [90] proposed an algorithm for the SAM robot to locate and bring objects into the home as part of the ARMEN project. First, the SURF algorithm is used to extract the features. Then, an SVM algorithm is implemented to classify the objects. Another example is presented by McMullen et al. in [91]; they proposed an algorithm for the HARMONIE system that consists of a robotic arm, a Kinect sensor, and EGG control to help disabled people to pick up objects. The authors used the Point Cloud Library to segment spherical objects. Then, the largest planar surface was computed to determine the location of the objects. Finally, this information was used to move the robotic arm to the desired object location. In addition, Loconsole et al. in [92] proposed an algorithm for an assistive skeleton robot to be able to help patients to move their arm during rehabilitation. The authors implemented a Kinect sensor to extract 2D and 3D images. First, during 2D processing, the skin points and background are removed. Then, during 3D processing, the Viewpoint Feature Histogram (VFH) algorithm is implemented to detect and track the cylindrical object. Finally, the location of the object is used to move the skeleton. Additionally, Quintero et al. in [93] performed the Vision-Based Interface (VIBI) algorithm for the JACO robot capable of aiding upper-body-disabled people in picking up objects. The authors implemented a Kinect sensor to obtain a point cloud. Then, Random Sample Consensus (RANSAC) and a 2D convex hull are used to determine the plane coefficients, inliers, and points that belong to the table. Next, the distances of the inliers are calculated to group them and distinguish the objects. Finally, the mean vector for each cluster and the minimum bounding box are calculated.

Similarly, Jain and Argall in [94] proposed an algorithm for the Mico robot arm to recognize everyday objects in the house. First, the Kinect sensor is implemented to extract a point cloud to analyze the surface and approximate the object’s geometry. Finally, the RANSAC algorithm is used to select a primitive geometry for each point cloud cluster. Another project based on the JACO robot is presented by Bousquet et al. in [95]. The authors performed an algorithm to recognize and pick up objects on a table. First, a Kinect sensor is implemented to extract a point cloud. Then, a backpropagation neural network is used to segment the objects through the RGB-D texture (colors and spatial positions). Finally, the point cloud of each object is analyzed to eliminate outliers and determine their locations. Moreover, Gualtieri et al. in [96] presented an algorithm for the Baxter robot capable of offering aid to disabled people to pick up everyday objects. The authors implemented an RGB-D camera to extract a point cloud and detect objects by employing a deep convolutional neural network (DCNN). Then, the geometry of the object is calculated, enabling the robot to pick it up.

Furthermore, Zhang et al. in [97] proposed an algorithm for an assistive robot to help disabled people to drink. The authors used a Kinect sensor to obtain a 3D image and a brain–machine interface to monitor drinking intention. They implemented a plane extraction algorithm for background subtraction. Next, the convex hull searches and region growth (RG) algorithms were performed to segment the objects. Finally, a CNN was applied to recognize the object and for the robot to pick it up and bring it to the person’s mouth.

In addition, Erol et al. in [98] presented an algorithm for the Turtlebot 2 robot to be able to recognize objects during a navigation task in a home environment. First, a CNN is used for the detection of multiple objects to group them. Then, the most representative points of each object are extracted to store them in a database and use to recognize places in a home. On the contrary, a simulated option is presented by Wang et al. in [99] for an assistive robotic arm capable of helping disabled people to pick up objects on the floor. The authors implemented an RGB-D camera to extract cloud points from the scene. A Faster R-CNN and Inception V2 algorithm are used to detect and locate the objects; thus, the robotic arm can move to take them.

Moreover, Ka et al. in [100] proposed the assistive robotic manipulation assistance (AROMA) algorithm for the JACO robot capable of offering aid to pick up objects from a table. The authors adopted the Senz3D 3D camera to extract a point cloud from a scene. Then, the segmentation and moving average filtering on the RGB-D image were implemented, utilizing the alpha module from OpenCV 3.0.

Additionally, Ivorra et al. in [101] proposed an algorithm for the JACO 2 robot to help disabled people to pick up objects. The authors implemented the YOLO framework to detect all objects on a table. Then, the LINEMOD algorithm was used to locate the objects. Finally, this information was combined with the position of the eyes to manipulate the robot arm. Moreover, Kasaei et al. in [102] proposed the incremental multiple-object recognition and localization (IMORL) algorithm for the JACO 2 robot to offer aid to disabled people to pick up objects. First, they extract the point clouds employing Kinect. Then, they separate the objects within a cubic, and for each group of points, the key points are extracted. Finally, each object is represented as a histogram to compare it in a database and thus recognize the object. Furthermore, Shim et al. in [103] suggested a hybrid algorithm for an assistive robot capable of recognizing and picking up objects from both patients and healthy people. First, the authors use a brain–machine interface to detect the user’s intention to pick up a given object. Then, the Kinect sensor is implemented to detect, recognize, and locate the object using YOLO. Finally, the robotic arm calculates the movement to pick up and carry the object.

For SARs, object detection is crucial because it improves the human–robot interaction. The algorithms found in the literature are varied. However, authors have chosen to implement those based on 3D images and remove the surface where they are placed by employing point cloud clustering. Another critical point is that most SARs that recognize objects have an ARM system incorporated, making them capable of manipulating objects (see Table 3).

### 4.2. Algorithms Used to Detect the Environment

The detection of the environment for SARs is essential when navigating at home or at the workplace to aid users with their ADLs. This is not straightforward because objects have different features and can be in different positions or locations. Next, the algorithms used for the recognition of the environment implemented by SARs are presented.

One example of such an algorithm is presented by Meng et al. in [104], who proposed an algorithm for an assistive robot capable of navigating in indoor environments. The authors implemented a Kinect sensor to obtain a depth image. Then, the Oriented FAST and Rotated BRIEF (ORB) algorithm from OpenCV was used for feature extraction and description. Next, the RANSAC algorithm was applied to remove outliers. Finally, the locations and edges of the objects were computed to represent complete 3D models utilizing the OctoMap framework. Additionally, Furuta et al. in [105] proposed an algorithm for the PR2 robot that is capable of recognizing and navigating in domestic environments. The authors used a Kinect sensor to extract an RGB image and point clouds to calculate the bounding boxes of objects. Then, an FCNN was implemented to classify the objects in a refrigerator, table, drawer, door, and background. This information was used for navigation planning. Additionally, Papadakis et al. in [106] proposed the projective light diffusion imaging process (PLDI) algorithm for the ROMEO2 robot capable of recognizing domestic objects for navigation planning. The authors implemented joint bilateral filtering to reconstruct the RGB-D images and ignore pixels with invalid depth values. Then, the orientation of the objects was calculated using the 3D surface orientation tensor. Next, the points of the objects were clustered and separated from the ground utilizing the RANSAC algorithm. Finally, the panoramic object representation for accurate model attributing (PANORAMA) algorithm was used to classify the 3D objects. Nevertheless, Nagahama et al. in [107] developed the shape manipulation model estimator (SVE-V) algorithm for Toyota’s human support robot to recognize and open a door in domestic environments. The authors used an RGB-D sensor to extract a point cloud and calculate all planes perpendicular to the floor surface. Next, the Canny algorithm was implemented to detect edges on the 2D plane. Finally, the Hough transform was employed to detect the positions of all doors. This information was used to calculate the movement of the robot arm.

On the other hand, Othman et al. [108] proposed an algorithm for the NAO robot to be able to detect whether a door is open or closed. The authors first perceived the environment with a 2D camera. Then, a CNN was implemented to detect the doors. Finally, social robot indoor navigation (SRIN) classifier was used to build a map and determine the door’s state. This information was used for the robot’s navigation module.

SARs must not only recognize small objects to bring them to people. In the literature, it has been found that assistive robots must have the ability to recognize the environment. Most of the authors implemented algorithms based on 3D images for mapping and locating robots. In addition, it was found that the RANSAC algorithm is the most used to remove the floor (see Table 3).

In this section, the algorithms used with SARs to recognize objects were introduced. Table 3 shows a comparison between these algorithms, and it can be consulted for authors interested in implementing them, improving them, or developing a new one in designing an SAR.

**Table 3:** Comparison of object algorithms.

Algorithm	PERF	Speed	Hardware and Software	Application
ARMAR-III (2010) [82]	-	-	ARMAR-III robot with a DSP56F803 from Motorola and a FPGA EPF10k30a from Altera	Objects
FRIEND (2011) [86]	-	offline	FRIEND robot and PC with a 1 GHz Intel-Processor	Objects
ORC (2013) [88]	-	offline	Not described	Objects
SIFT (2013) [89]	93.650%	real time	Pioneer 3-DX mobile robot	Objects
SURF (2013) [90]	-	-	SAM robot	Objects
3D (2013) [91]	77.8%	real time	Robotic arm, a Kinect and EGG sensors	Objects
ORB & RANSAC (2014) [104]	-	-	Not described	Environment
VFH (2014) [92]	-	real time	Workstation with Intel Core2 Quad Q9550 processor, 3 GB RAM, and Windows 7	Objects
VIBI (2015) [93]	-	-	JACO robot and Kinect sensor	Objects
MICO (2016) [94]	82%	real time	Mico robot arm and Intel Core i7 Quad-Core processor PC with 12 GB of RAM, and Ubuntu 12.04	Objects
AROMA (2016) [100]	85%	real time	JACO robot and Senz3D 3D camera	Objects
FCNN (2017) [105]	100%	on line	PR2 robot and Kinect	Environment
Backpropagation (2017) [95]	95%	real time	JACO robot and Kinect, 3.60 GHz Intels CoreTM i7-4790 CPU, 16 GB of RAM, and an NVIDIA GeForces GTX 760 GPU	Objects
Mixed (2017) [83]	96.1%	real time	NVIDIA GeForce GTX 745. It includes 384 Compute Unified Device Architecture (CUDA) cores with 4 GB memory	Objects
DCNN (2017) [96]	90%	real time	Baxter robot	Objects
RG & CNN (2017) [97]	99.05%	real time	Not described	Objects
SME-V (2018) [107]	91.7%	-	Toyota’s Human Support Robot, Xtion PRO LIVE, an RGB-D camera	Environment
FastOrient (2018) [84]	91.1%	-	Universal Robots UR10 robot and Matlab	Objects
PLDI (2018) [106]	73.5%	real time	ROMEO2 robot and Workstation with i7 Intel processor	Environment
CNN (2018) [98]	-	real time	Turtlebot 2 robot and a workstation with an Intel i5 and a NVIDIA GTX-1080 with 8 GB of memory	Environment
YOLO (2018) [101]	94.4%	real time	JACO 2 robot and a workstation with Intel i7 processor, 8 GB of RAM and a Nvidia GeForce GTX680 GPU	Objects
IMORL (2018) [102]	79%	real time	JACO 2 robot	Objects
YOLO & BMI (2019) [103]	78%	real time	Not described	Objects
Martinez (2019) [85]	97.5%	real time	Baxter robot, the Pepper robot, and the Hobbit robot	Objects
Faster R-CNN (2019) [99]	78.35%	21.62 FPS	Workstation whit CUDA enabled NVIDIA Tesla K80 GPU, and 8 GB RAM. Simulated on Virtual Robotics Experimentation Platform	Objects
CNN & SRIN (2020) [108]	97.96%	real time	NAO Robot	Environment

Performance (PERF) is the percentage of frames that have followed a face correctly. Speed is the number of FPS that can be processed. Hardware is the computer setup that was used to perform the tests. Software is the development platform used. The application refers to the task for which the algorithm is intended. Where data were not available, this is represented by a (-).

For algorithms for object recognition, the one proposed in [97] has the best performance reported (99.05%); it implements convex hull searches and region growth in conjunction with a CNN. Unfortunately, the authors did not report the hardware and software equipment employed. On the other hand, in [85], the authors reported performance of 97.5% using simple algorithms. In this case, the authors implemented their proposal in three different robotics platforms, and all of them run in real time.

Regarding environment recognition, 3D images are the most popular solution. In the literature, it has been found that the best performance (100%) is presented by [105] using an FCNN implemented in the PR2 robot with a Kinect sensor. The authors indicated that their tests were conducted in real time. In contrast, other authors did not report complete characteristics.

## 5. Discussion and Conclusions

In this paper, algorithms for face, body, and object recognition implemented by SARs were presented. It can be found in the literature that some authors coincide or proposed similar algorithms for performing the same tasks. In contrast, others decide to innovate and present new algorithms. However, for SARs, the performance and speed at which they are executed are important.

First of all, the traditional computer vision algorithms have some advantages, such as using few computer resources (RAM, no GPU). As noted in the Table 1, Table 2 and Table 3, articles published between 2010 and 2015 used conventional computers to deploy their algorithms. Moreover, it was found that these algorithms could be executed in real time, and some have performance above 90%. Although these traditional algorithms were the first to be developed, they are still an option when the user needs to perform an algorithm in an embedded or low-budget system. Even in 2019, Martínez et al. [85] used traditional algorithms to detect objects; therefore, the use of these should be considered in the development of SARs because some require more than one task to be performed at the same time, and the use of traditional algorithms helps to maintain performance and speed.

Based on the activity to be carried out, the following process helps readers to select an algorithm: (a) define the aspect of interest (face, body, or object localization) to select an algorithm from those shown in Table 1, Table 2 and Table 3; (b) identify the subject of interest; (c) prioritize requirements (velocity, performance, hardware, or software) (Usually, the response speed is the priority since SARs must interact with users naturally. In addition, the selected algorithm should have acceptable performance and execute the highest possible FPS with the minimum hardware and software resources.); (d) implement the selected algorithm.

In Section 2, some algorithms have been described. These algorithms allow SARs to interact with people through their faces. Of the 33 algorithms in Table 1, 19 are used to recognize facial expressions. The performance of these algorithms is between 72.42% and 92.26%. However, not all algorithms have specified that they run in real time. For instance, refs [30,36] were tested offline. Regarding the computer resources used to recognize facial expressions, two implementations are reported using basic hardware computers [34,39] and computers with graphic cards [30,31]. It is worth noting the use of robotic platforms such as the NAO [27,28,33,38], R-50 Alice [35,40], Pepper [32], kiwi [44], and N-Maria [42].

Similarly, in Table 1, nine algorithms used to follow the human face are discussed, whose performance ranges from 73.3% to 99%. These algorithms can be executed in real time using computer equipment with basic hardware characteristics [8,12,17,39] or robotic platforms such as the NAO robot [19]. Five algorithms used for facial recognition tasks are discussed, showing a performance range between 90% and 91%. The execution time of these algorithms has been specified as real time, and to achieve this performance and speed, computer equipment with basic hardware [21,25] and robotic platforms such as InMoov [22], Pepper [23], THIAGo [24], and ROBCO 20 [25] have been used.

On the other hand, in Section 3, the algorithms used for SARs to perform tasks involving the human body have been presented. In Table 2, 31 different algorithms are reported; 10 of these have been used in such a way that SARs can interact with people, and their performance ranges from 50% to 99.73%. Most authors consider it unnecessary to describe the computer hardware used [66,68,71,73,74]. However, in Table 2, it can be observed that computer equipment with basic hardware has been used [63], as well as robots such as FACE [67], Robovie R3 [69], and Pepper [70].

In addition, nine algorithms were used to monitor people’s exercise. The performance achieved by these algorithms ranges from 95% to 99.87%. Regarding the execution speed, all run in real time. For their implementation, computer equipment with basic hardware [52,57,61] has been used, as well as the robotic platforms NAO [55], Pepper [57], and COREAS [59].

Moreover, seven algorithms from Table 2 were used to monitor people’s states, mainly to detect falls. The performance of these algorithms is between 93% and 97.4%. Regarding the speed, one has five FPS [81] and another 24 FPS [75]. The rest runs in real time. In terms of computer resources, a computer with advanced hardware is used in [76], as well as robotic platforms such as MOBOT [77], Hobbit [80], Roomba [81], and a customized robot [78].

Additionally, five algorithms used to determine the activity carried out by a person are presented in Table 2. Their performance varies between 80% and 98.11%. Regarding the speed, these algorithms have been executed in real time at 20 FPS [51]. For their implementation, computers with basic [51] and advanced [50] hardware were used, as well as the robotic platforms Brian [47] and Pepper [49].

The algorithms used to determine a person’s activity and the algorithms to interact with SARs have similar functions to detect the parts of the body. In the same way, the algorithms designed to help people with their exercises are based on determining the positions of body parts. These algorithms are similar to those used to monitor a person as they are based on determining the posture and orientation of the whole body.

Section 4 describes the algorithms that allow SARs to recognize and interact with objects. Twenty-six algorithms are reported in Table 3. Among these, 20 have been employed to enable SARs to recognize and manipulate objects. The performance of these algorithms ranges from 77.8% to 99.05%. Regarding speed, it is shown that some algorithms were tested without considering the execution time [86,88], one algorithm is reported to run at a speed of 21.62 FPS [99], and the rest run in real time. Regarding hardware, the algorithms for object recognition were tested on robotic platforms such as the ARMAR-III robot [82] or the Jaco robot [93,95,100], to mention a few.

On the other hand, the remaining six algorithms in Table 3 were utilized for SARs to perceive the environment in which they are located. The performance range of these algorithms is from 77.8% to 100%. Additionally, it can be observed that these algorithms are executed in real time. Regarding hardware characteristics, robotic platforms such as the following robots were used: PR2 [105], Toyota’s Human Support [107], Romeo 2 [106], Turtlebot 2 [98], and NAO [108].

These algorithms are distinguished because 89% employ 3D images (see the objects section in Figure 1), which allows the identification of shapes and surfaces. However, it is also worth noting that they have been tested on robotic platforms performing physical interaction with objects, such as taking an object after recognizing it or opening a door.

Table 1, Table 2 and Table 3 report the performance of the algorithms. In particular, 43% of them are above 90% and, in some cases, close to 100%. For example, tracking the face with the HSV & RGB algorithm [18] offers performance of 99%; monitoring the exercise routine of a person with the CNN & LSTM algorithms [57] yields performance of 99.87%.

In Table 1, Table 2 and Table 3, the execution times are reported. For example, the DOF & CNN 2D algorithm [80] runs at five FPS. On the other hand, the algorithm proposed in [23] YOLO & FaceNet runs at 30 FPS. Moreover, some authors specify that their algorithm can work in real time without specifying the number of FPS, such as the HOG, SVM & HSV algorithm proposed in [79]. However, the speed needed to run an algorithm depends on the task to be performed. When a task for human motion detection is being performed, running an algorithm at 10 FPS can be considered real-time [109], since it is sufficient to detect motion changes between frames without losing continuity.

Table 1, Table 2 and Table 3 describe the hardware specifications required to implement the algorithms. These specifications can consider essential characteristics or computers with graphics cards that significantly improve any algorithm’s execution speed. For example, to implement an algorithm that determines facial expressions, one could choose between the DBN algorithm [38] (which requires a computer with an i7 processor and 4 GB RAM) and YOLO & FCNN algorithm [31] (which requires a computer with an i7 processor and at least an Nvidia Tesla K80 graphics card). Both algorithms run in real time, but the first one does not indicate its performance, while the second one had a performance of 72.47%. Finally, to choose the algorithm to be used, it is recommendable to consider the hardware requirements for its implementation. In this case, the DBN algorithm [39] is implemented using basic image processing. It requires less computational power than the YOLO & FCNN algorithm [31], which requires a deep neural network execution.

Regarding the new algorithms, these are robust to disturbances, mainly to occlusions. In addition, in the case of 3D images, the errors that traditional algorithms present during detection and location can be corrected. Most authors implement neural networks because they have a performance above 95%. In this way, some frameworks, such as OpenPose and Yolo, based on NN, have been developed and used. On the other hand, to execute these algorithms, authors report that it is necessary to use computers with resources greater than standards (RAM and slow processors) and video graphic boards with GPU. Some other authors prefer the use of robotics platforms that already integrate these resources. However, this causes the development of SARs to increase in price.

For SARs, the algorithms run in real time because they must react to human actions, ensuring that the human–machine interaction is as natural as possible. The algorithms executed in real time do not necessarily process at the same number of FPS; this depends on the application. For SARs, it is enough to track the movement constantly, avoiding the loss of sequences of frames that hinder the algorithm. Although most cameras record at 30 FPS, some algorithms can work in real time below this rate.

Some authors did not report the complete information (performance, speed, hardware, and software). However, their research projects are important because they are part of the literature, and authors interested in algorithms for SARs can use the information as background. However, it is recommended that authors report all variables that influence their results in order to allow others to select one algorithm over another.

Finally, for authors who wish to develop SARs, it is recommended to establish the system and user requirements in order to determine the appropriate algorithm for their project. Although more sophisticated algorithms such as deep learning have become popular due to their robustness, this does not justify disregarding traditional algorithms since they do not have high computational requirements. It is even a viable option to combine both so that an SAR can perform multiple tasks with different computational resources.

## Data Availability

We did not report any data.

## Abbreviations

The following abbreviations are used in this manuscript:

ADL	Activities of Daily Living
AKM	Angular Kinematics Model
ANN	Artificial Neural Network
AO	Adam Optimizer
ASD	Autism Spectrum Disorders
ARMs	Assistive Robotic Manipulators
AROMA	Assistive Robotic Manipulation Assistance
cGAN	Conditional Generative Adversarial Network
CLNF	Conditional Local Neural Fields algorithm
CNN	Convolutional Neural Network
CLUE	Coded Landmark for Ubiquitous Environment
DBN	Dynamic Bayesian Network
DCNN	Deep Convolutional Neural Network
DOF	Dense Optical Flow
FCNN	Fully Convolutional Neural Network
FED	Facial Expression Detection
FPS	Frames Per Second
GMM	Gaussian Mixture Models
GPU	Graphics Processing Unit
HOF	Hall-of-Fame
HOG	Histogram of Oriented Gradients
HMM	Hidden Markov Model
HSV	Hue, Saturation, Value
IMORL	Incremental Multiple-Object Recognition and Localization
KLT	Kanade–Lucas–Tomasi
KNN	K-Nearest Neighbors
LBPH	Local Binary Pattern Histogram
LDA	Latent Dirichlet Allocation
LSTM	Long Short-Term Memory
MBH	Markov–Block–Hankel
MCI	Mild Cognitive Impairments
MMDO	Maximum Margin Object Detection Model
MOG	Mixture of Gaussians
MTCNN	Multi-Task Cascade Convolutional Neural Network
MT-DNN	Multi-Task Deep Neural Network
ORC	Optical Character Reader
PANORAMA	Panoramic Object Representation for Accurate Model Attributing
PCA	Principal Component Analysis
PLDIP	Projective Light Diffusion Imaging Process
RANSAC	Random Sample Consensus
RBF-NN	Radial Basis Function Neural Network
RF	Random Forest
RGB	Red, Green, Blue
RGB-D	Red, Green, Blue and Depth
SA	Scene Analyzer
SARs	Socially Assistive robots
SDALF	Symmetry-Driven Accumulation of Local Features
SDF	Signed Distance Functions
SDM	Supervised Descent Method
SIFT	Scale-Invariant Feature Transform
SRIN	Social Robot Indoor Navigation
SURF	Sped-Up Robust Features
SOM	Self Organizing Map
SVM	Support Vector Machines
TBI-FER	Traumatic Brain Injured Facial Expression Recognition
VFH	Viewpoint Feature Histogram
VIBI	Vision-Based Interface
VJ	Viola–Jones
YOLO	You Only Look Once
